# Is Crime a Disease

**Published:** 1890-09

**Authors:** 


### IS CRIME A DISEASE.

In recent times the idea has become very prevalent among a certain class of sentimentalists that crime is not an indication of innate depravity, but a symptom of mental and physical disease, or an inheritance from some criminal ancestor, and, therefore, the much abused murderer or robber is not a bad man, but only an unfortunate one, deserving of pity and care rather than punishment. A sick man, they say, ought not to be imprisoned, and one who is staggering under a load of homicidal tendencies bequeathed to him by his grandfather, is not worthy of death, even if he does occasionally send some of his less unfortunate fellow beings into the next world.

To a certain extent, the above ideas are correct; a man physically and mentally sound is less likely to commit criminal acts than one with a diseased body or of abnormal mental action, and the history of the notorious Jukes family proves beyond question that from a single criminal ancestor may spring a long line of descendants, a majority of whom will be enemies to the welfare of society. But, granting that a tendency to crime may be inherited, it must originate somewhere, and if a man's ancestor may have been a spontaneous criminal as it were, another man of the present day may also be laying up, on his own responsibility, a heritage of crime for his unborn descendants.

It has been suggested—and the plan has met with general approval—that criminals should not be allowed to marry and reproduce their kind to the injury of posterity. This would be a most excellent method of reducing the criminal population, if the prohibition of a legal marriage to such persons would ensure their leaving no descendants. Unfortunately, the victims of "atavism" have inherited a tendency to look upon the marriage laws of modern society as superfluous, and we fear that the total number of children born among them would not be very greatly reduced by any such enactment.

Practically, it is of very little consequence whether crime is a disease, an inheritance, or an original manifestation. The criminal is an enemy to society under any circumstances, and society has a right to protect itself from him. If a man is not able to live among his fellows without robbing them or otherwise injuring their property or persons, let him be removed from among them and permanently confined where he can do them no harm. It is not a question of punishment or revenge, but of self protection. If the abnormal tendencies can be eradicated, and the criminal made a useful member of society, every effort should be made to that end; but if crime is a disease, it seems—at least in its more serious manifestations—to be an incurable one. The percentage of reformed criminals is discouragingly small, and that of those convicted for subsequent offences disproportionately large. The best treatment of such persons is a perplexing question, but the right of self preservation is paramount to every other consideration, and the morbid, unwholesome sympathy shown by an increasing class of people towards those who are so much out of harmony with their social environment, will only result in great injury to the orderly and law abiding classes of society, without causing any decrease of crime or conferring any permanent benefit upon the criminals themselves.

